# Chicago Medical Society

**Published:** 1889-04

**Authors:** 


*Stated Meeting, February 4.*

THE PRESIDENT IN THE CHAIR.

DR. C. T. PARKES reported some cases of

SKIN GRAFTING BY THIERSCH'S METHOD.

Mr. A. Wielberg, age 48, came to the clinic in Rush Medical College January 19th, with a circular sarcoma of the right side of the head and face, measuring three inches in diameter and one and a half inches in thickness. The tumor was slightly movable, and had ulcerated throughout its superficial surface; had an angry appearance, and bled profusely upon slight manipulation. The patient had first noticed it five years ago. It had grown slowly at first, but more rapidly within the last few weeks. The face and scalp were very thoroughly disinfected, the scalp shaven, first being thoroughly washed with soap and water, then with clean water, then with bichloride of mercury, $\frac{1}{2000}$. The tumor was removed by a circular incision one-half inch from its edge on all sides; then it was dissected up, together with a portion of the temporal muscle. Then the zygoma and part of the malar was chiseled away, and almost all the tissues removed down to the temporal parietal and molar bones and upper part of parotid gland to the extent each one had been covered by the tumor. The vessels were ligated and the wound irrigated and dressed antiseptically. For want of time the wound was not covered with skin at this time, but was permitted to granulate, which process went on in a perfectly normal and aseptic condition for ten days. I now proceeded to transplant skin on this surface by the method introduced by Prof. Thiersch.

This case was grafted one week ago, and now you see the entire surface is covered with skin. Without this treatment it would have taken this surface several months to heal.

In another case a child 8 years old had received enormous wounds from burns, almost completely covering one hand and the postérior surface of one thigh. These ulcers had been treated for several months without much benefit, and the child's leg was becoming more and more flexed at the knee. In one week each of these ulcers was covered with skin, and now the child, which was an emaciated cripple, looks fat and rosy, and has regained the use of its leg.

In a young man with atresia of one nostril, an opening was made and lined with skin, held in place by means of a plug of iodoform gauze covered with vaseline, and the patient dismissed with a good nostril two weeks later.

In another case the grafts became infected in some manner, and three-fourths of them were destroyed by the activity of the pus microbes.

Using no antiseptic solutions in dressing these wounds, it is evident that the greatest care must be taken to have most absolutely aseptic conditions preserved, for these delicate grafts are not able to compete with the microbes of suppuration in the struggle for existence, until they have gained a firm foothold. The method saves months of time to the patient, and hence money; enables him to get to work much earlier, and, too, saves the surgeon much annoyance. If irritation has anything to do with the quick return of malignant, this method certainly will diminish such danger. Again, as the grafts can usually be obtained from the patient, the danger of inoculation of tuberculosis and other troubles, likely to occur after transplantation from other persons, is entirely avoided.

